# Preparation of MoS_2_-based polydopamine-modified core–shell nanocomposites with elevated adsorption performances

**DOI:** 10.1039/c8ra02964d

**Published:** 2018-06-13

**Authors:** Shuxin Sun, Tifeng Jiao, Ruirui Xing, Jinghong Li, Jingxin Zhou, Lexin Zhang, Qiuming Peng

**Affiliations:** State Key Laboratory of Metastable Materials Science and Technology, Yanshan University Qinhuangdao 066004 China tfjiao@ysu.edu.cn; Hebei Key Laboratory of Applied Chemistry, School of Environmental and Chemical Engineering, Yanshan University Qinhuangdao 066004 China zhanglexin@ysu.edu.cn; State Key Laboratory of Biochemical Engineering, Institute of Process Engineering, Chinese Academy of Sciences Beijing 100190 P. R. China

## Abstract

New molybdenum disulfide (MoS_2_)-based core–shell nanocomposite materials were successfully prepared through the self-assembly of mussel-inspired chemistry. Characterization by Fourier transform infrared, thermogravimetric analysis, scanning electron microscope and transmission electron microscopy revealed that the surface of the flaked MoS_2_ was homogeneously coated with a thin layer of polydopamine (PDA). Dye adsorption performances of the synthesized MoS_2_–PDA nanocomposites were investigated at different pH values and reaction times. Compared with pure MoS_2_ nanosheets, the obtained core–shell nanocomposites showed elevated adsorption performances and high stability, indicating their potential applications in wastewater treatment and composite materials.

## Abstract 2

New core–shell MoS_2_–PDA nanocomposites are prepared *via* mussel-inspired chemistry and a simple interfacial self-assembly process, demonstrating potential applications in wastewater treatment and self-assembled core–shell composite materials.

## Graphical abstract

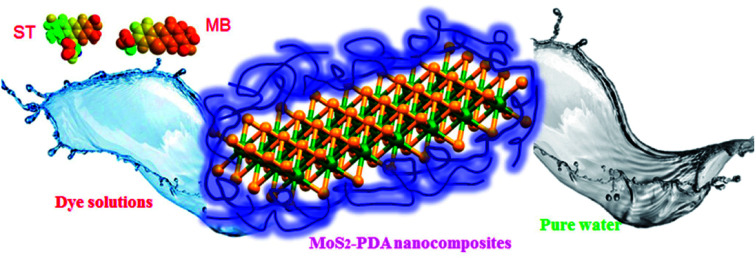

## 1. Introduction

Over the past several decades, graphene has been attracting a great deal of attention due to the unusual properties associated with its ultrathin structure.^1–7^ Among other two-dimensional layer materials,^8–11^ molybdenum disulfide (MoS_2_) is one of the most attractive and has a similar structure to graphite.^12–16^ MoS_2_ has a sandwich structure consisting of two layers of sulfur atoms and a middle layer of molybdenum atoms, which are bonded by van der Waals forces.^17–20^ MoS_2_ nanosheets have many excellent physical and chemical properties, and demonstrate important applications in sensors,^21–24^ optoelectronic devices,^25,26^ catalysis^27,28^ and other fields.^29^ In addition, MoS_2_ nanocomposites have attracted wide study in the field of polymer nanocomposites.^30–33^ For example, Li *et al.* investigated polyethylene glycol (PEG)-modified MoS_2_ surfaces,^34^ and the obtained products showed good removal efficiency toward some dyes.

In recent years, mussel-inspired chemistry has become a hot research topic in materials science, chemistry and other fields.^35–37^ In the marine environment, mussels can secrete proteins through their feet, which have excellent adhesion and good biocompatibility. Dopamine, as an imitation mussel-protein material, has strong adhesion properties through a complex self-assembly process to form a polydopamine (PDA) coating with various functions.^38^ The formed PDA layer can be used to modify the surface of inorganic and organic materials, so it demonstrates wide prospects for application in the fields of separation membranes, adsorbent materials, biomedical materials, biological binders and so on.^39–42^ Now core–shell materials have shown great potential applications in biology, electricity, catalysis and so on.^43–46^ For example, Zhao *et al.* synthesized core–shell diamond-based nanocomposites, exhibiting high activity and high catalytic performance.^47^ Although the catalytic performance of prepared composite materials may seem ideal, there are still some deficiencies, such as high cost and harsh operating conditions. Thus, mussel-inspired chemistry has the advantages of mild reaction conditions, a wide range of applications and diverse functions, *etc.* On the other hand, core–shell nanocomposites have unique structural characteristics, integrating the properties of both internal and external materials.

In this work, we used PDA to modify the surface of MoS_2_ nanosheets to synthesize core–shell nanocomposites. The as-prepared composites were characterized by a series of morphological and spectral characterization techniques. The results showed that we had successfully prepared MoS_2_–PDA polymeric materials. The obtained MoS_2_–PDA composites were used as adsorbents for the removal of methylene blue and Safranine T, and they showed enhanced adsorption ability.

## 2. Materials and methods

### 2.1 Materials

Molybdenum disulfide (MoS_2_, 99.9%) nanosheets were obtained from Huajing Powdery Material Science and Technological Co., Ltd. (Hunan, China). Hydroxyphenethylamine hydrochloride (dopamine, 98%) and tris(hydroxymethyl)aminomethane hydrochloride (Tris–HCl, 99%) were purchased from Aladdin Chemicals and Alfa Aesar Chemicals (Shanghai, China). Methylene blue (MB) and Safranine T (ST) were obtained from Tianjin KaiTong Chemical Reagent and Sinopharm Chemical Reagent Co., Ltd. without further purification. Deionized (DI) water was used to prepare aqueous solutions in all experiments.

### 2.2 Fabrication of the MoS_2_–PDA nanocomposites

The synthesis of core–shell MoS_2_–PDA nanocomposite was undertaken according to the method given in previous literature.^48^ Briefly, 300 mg of MoS_2_ nanosheets were first dispersed in 100 mL of Tris buffer (10 mM, pH = 8.5) with sonication for 10 minutes, and then 200 mg of dopamine was added to the above solution with stirring. After stirring at room temperature in the dark for 12 h, 24 h, and 48 h, the obtained composite products were washed several times with water and ethanol, respectively, then centrifuged at 6000 rpm for 10 min. Finally, the products were freeze-dried for 2–3 days for the next experiments.

### 2.3 Adsorption performance test

In order to investigate the dye adsorption performances of MoS_2_ nanosheets and MoS_2_–PDA nanocomposites, we selected MB and ST as model dyes to complete the experiment. 10 mg of MoS_2_ nanosheets and 10 mg of the prepared MoS_2_–PDA nanocomposites were added to 100 mL of MB solution (8 mg L^−1^) and ST solution (30 mg L^−1^), respectively. After different adsorption time intervals, the samples were centrifuged and the supernatant analyzed by a UV-Vis spectrophotometer (664 nm for MB; 530 nm for ST). In addition, we also studied the effect of MoS_2_ and MoS_2_–PDA on MB adsorption with different initial solution pH values. The pH of the initial solution was adjusted to values of 2–11 by adding diluted aqueous HCl solution or NaOH solution.^20^ All experiments were carried out at room temperature under dark conditions. The amount of adsorbed dye per unit mass of adsorbent *q*_*t*_ (mg g^−1^) at time *t* (min) is calculated from the following formula:1
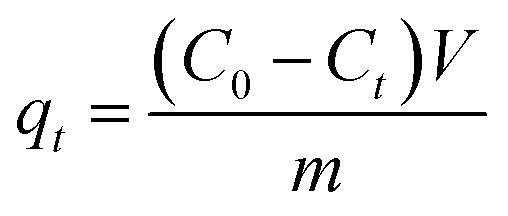
where *C*_0_ is the initial concentration of the adsorption solution (mg L^−1^), *C*_*t*_ is the concentration of the adsorption solution at time *t* (mg L^−1^), *m* is the total MoS_2_ sample added (g), and *V* is the volume of the adsorption solution (L).

### 2.4 Characterization

The microstructures of the samples were obtained using a field-emission scanning electron microscope (SEM) (S-4800II, Hitachi, Japan) equipped with an energy dispersive X-ray spectroscope (EDS) and a transmission electron microscope (TEM) (HT7700, Hitachi High-Technologies Corporation, Japan) with an accelerating voltage of 20 kV. Thermogravimetric analysis (TGA) was performed by a NETZSCH STA 409 PC Luxx simultaneous thermal analyzer (Netzsch Instruments Manufacturing Co, Ltd, Germany). BET measurements (NOVA 4200-P, US) were taken to characterize the specific surface areas and pore diameter distribution. Fourier transform infrared (FTIR) spectra were accomplished on a Thermo Nexus 470 FT-IR spectrometer (KBr disk). X-ray photoelectron spectroscopy (XPS) was performed using a Bragg diffraction setup (SMART LAB, Rigaku, Japan) with an Al Kα X-ray source. The adsorption experiments were monitored using a Shimadzu UV2550 spectrophotometer. All experimental processes were undertaken in a beaker under dark conditions. The synthesized composite materials were completely dewatered using an FD-1C-50 freeze dryer (Beijing Boli Experimental Instruments Co., Ltd., China). All aqueous solutions were prepared with water purified in a double-stage Millipore Milli-Q Plus purification system.

## 3. Results and discussion

### 3.1 Characterization of nanocomposites

**Fig. 1:**
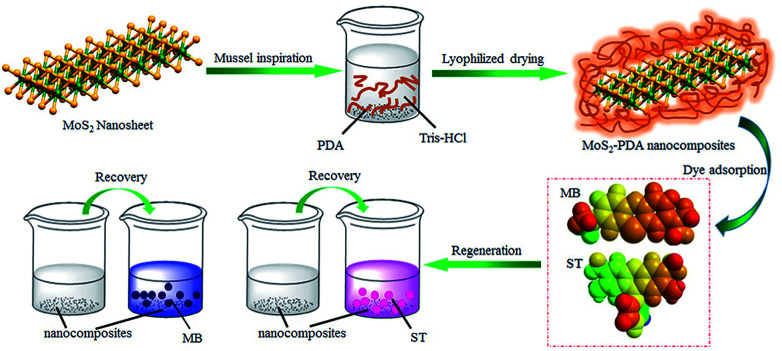
Schematic illustration of the fabrication of MoS_2_–PDA composites by mussel-inspired chemistry.

Firstly, Fig. 1 shows the entire experimental process, including the preparation of MoS_2_–PDA and the adsorption experiments of the obtained nanocomposites. First, MoS_2_–PDA nanocomposites were obtained by modification of MoS_2_ nanosheets with a PDA layer in Tris buffer solution and dried by centrifugation. Then two model dyes (MB and ST) were used to evaluate the adsorption capacity of the prepared composite materials.

**Fig. 2:**
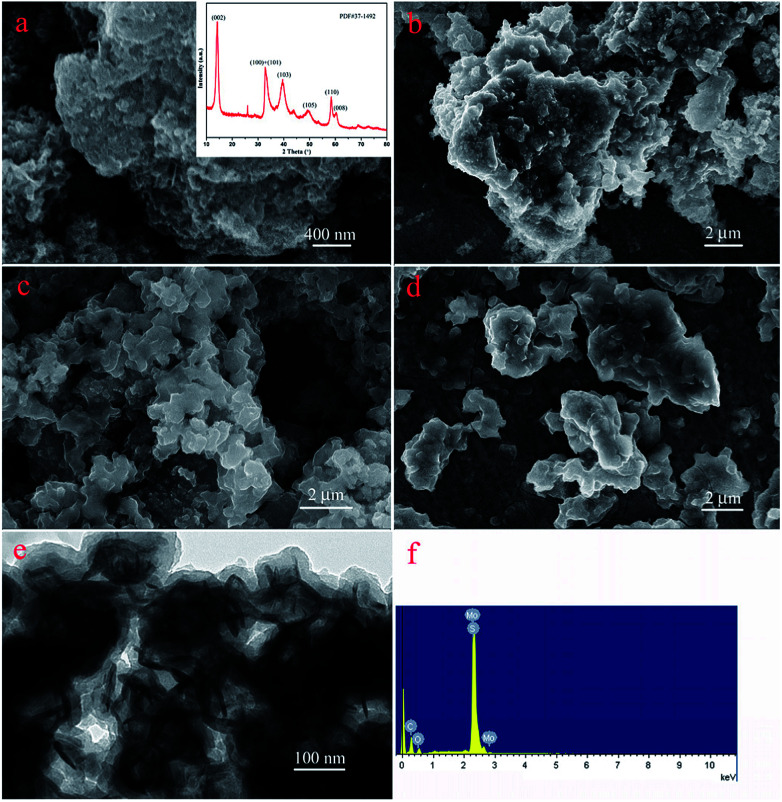
SEM and TEM images of as-prepared materials: (a) SEM image and inserted XRD data of MoS_2_; (b–d) SEM images of core–shell MoS_2_–PDA composites after different modification times (12, 24, and 48 h); (e) and (f) TEM image and EDX spectra of MoS_2_–PDA-48.

The nanostructures of the obtained materials were investigated by SEM and TEM, as shown in Fig. 2. Obviously, compared with the original MoS_2_ (Fig. 2a), with an increase in modification time, the layer thickness of PDA coated on the MoS_2_ surface increased and showed a tendency to aggregate together, as shown in Fig. 2b–d. In addition, Fig. 2e represents the TEM image of the MoS_2_–PDA composite with a modification time of 48 h, which was named MoS_2_–PDA-48. In the TEM image, it was obvious that the outer edge of the MoS_2_ nanosheets demonstrated some ultra-thin layer structures, which indicated that the PDA layer had been successfully anchored on the surface of the MoS_2_ nanosheets. Moreover, the typical XRD pattern of MoS_2_ (the inset image of Fig. 2a) and the EDX pattern of MoS_2_–PDA-48 (Fig. 2f) also provided obvious characteristics and suggested the above speculations.

**Fig. 3:**
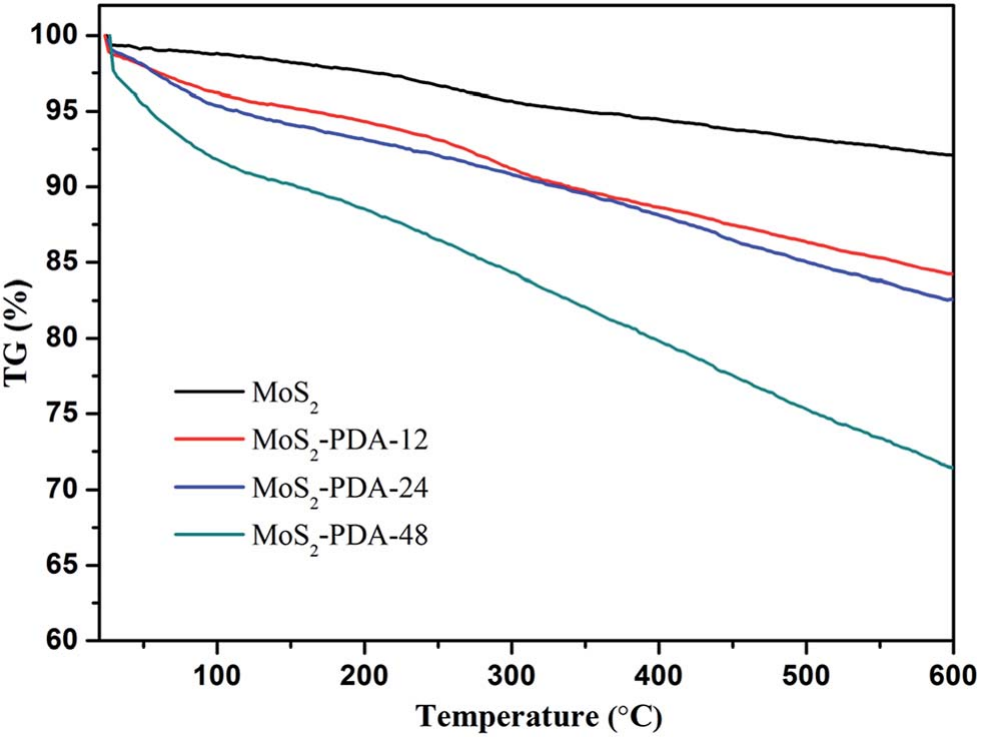
TG curves of MoS_2_ nanosheets and the obtained MoS_2_–PDA nanocomposites.

In addition, the thermal stability of MoS_2_ and the obtained MoS_2_–PDA composites were characterized and are shown in Fig. 3. According to the TG curves, MoS_2_ showed a low mass loss (about 7.9%) from room temperature to 600 °C, which indicated good thermal stability. The mass losses of the MoS_2_–PDA composites took the values of 15.8%, 17.4%, and 28.6% with different modification times (12 h, 24 h, and 48 h, respectively). The obtained results indicated that the PDA layer had been successfully coated onto the MoS_2_ surface and the amount of PDA component in the composites increased with an increase in modification time.

**Fig. 4:**
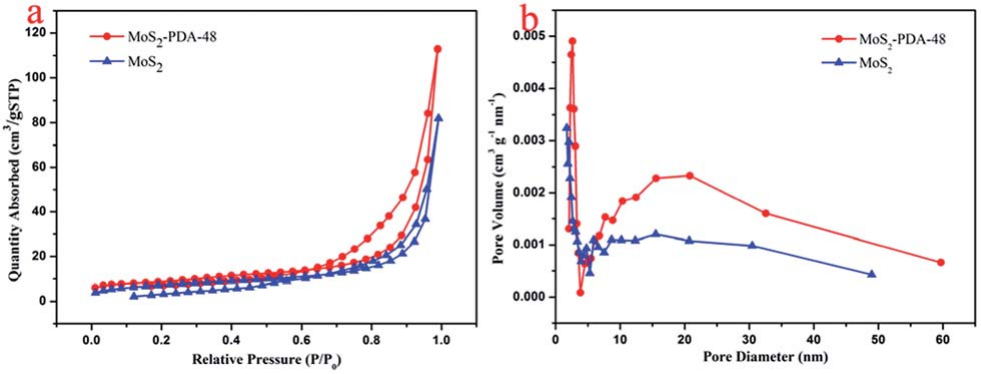
(a) Adsorption–desorption isotherms and (b) pore size distribution of MoS_2_ and the MoS_2_–PDA-48 nanocomposite.

In order to study the microstructure characteristics of MoS_2_ and MoS_2_–PDA-48, Fig. 4a shows the BET measurements using N_2_ adsorption–desorption isotherms. The adsorption–desorption isotherms of both MoS_2_ and MoS_2_–PDA-48 belonged to type IV isotherms. The hysteresis loop of MoS_2_ has an abrupt increase in the amount of adsorption at *P*/*P*_0_ = 0.6–0.95, which is characteristic of a mesoporous material. For the MoS_2_–PDA-48 nanocomposites, a hysteresis loop could be observed at pressure *P*/*P*_0_ = 0.8–0.95 that indicated a mesoporous structure. The pore size distribution curves were obtained according to the BET method and are shown in Fig. 4b. The pore size distribution of MoS_2_–PDA-48 showed a larger mesoporous size of 22.84 nm, compared to 19.62 nm for MoS_2_. The data for BET surface area and total pore volume are listed in Table 1. The specific surface area of MoS_2_–PDA-48 was calculated to be 30.57 m^2^ g^−1^, which was much larger than that of MoS_2_ (25.83 m^2^ g^−1^). The higher specific surface area can increase the number of active sites on the surface of the MoS_2_–PDA composite and enhance the chances of dye molecules anchoring at active sites, thus making adsorbents with better adsorption properties.

**Table:** Physical data of MoS_2_ and the MoS_2_–PDA-48 nanocomposite

Sample	Specific surface area (m^2^ g^−1^)	Average pore diameter (nm)	Pore volume (cm^3^ g^−1^)
MoS_2_	25.83	19.62	0.127
MoS_2_–PDA-48	30.57	22.84	0.175

**Fig. 5:**
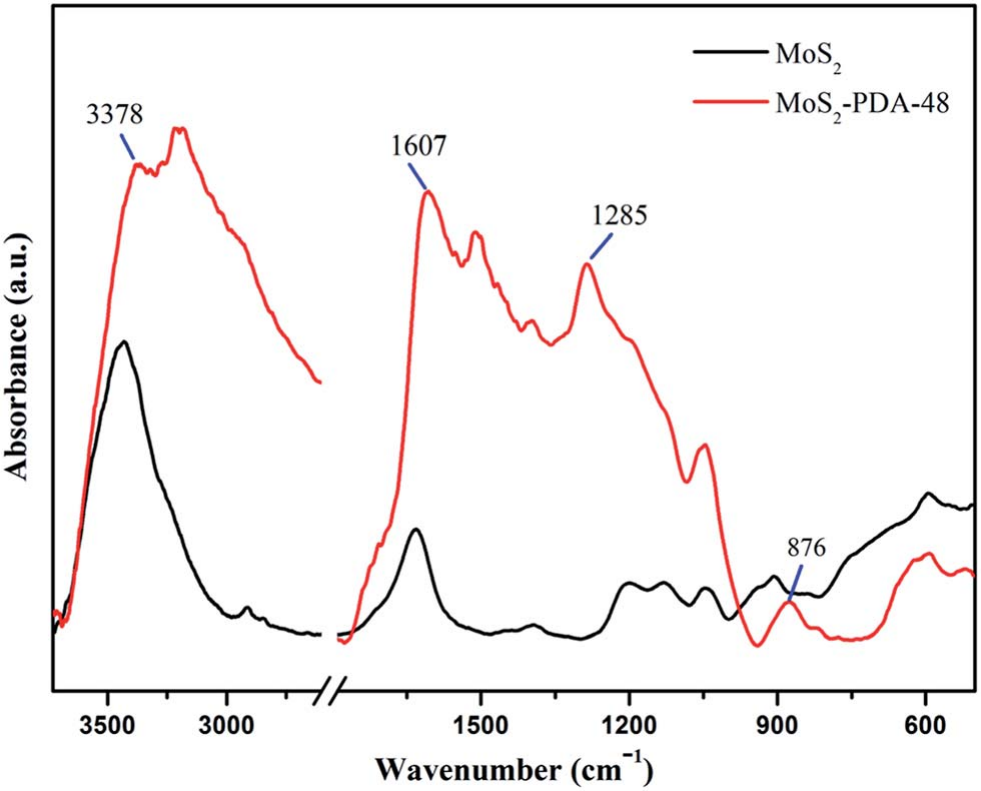
IR spectra of MoS_2_ and the MoS_2_–PDA-48 composite.

**Fig. 6:**
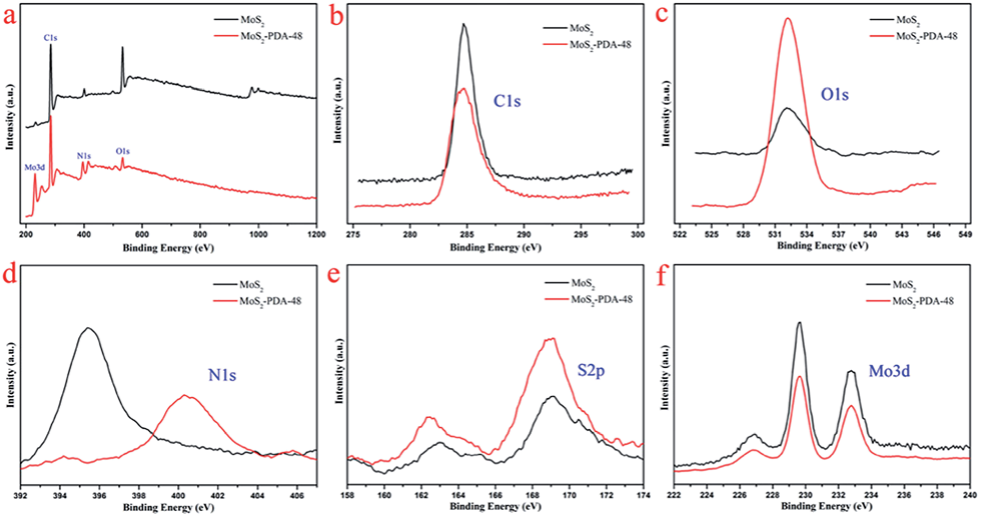
(a) XPS survey spectra of MoS_2_ and MoS_2_–PDA-48; high resolution scans of different elements: (b) C 1s; (c) O 1s; (d) N 1s; (e) S 2p; (f) Mo 3d.

FT-IR spectra of MoS_2_ and the MoS_2_–PDA-48 composite are demonstrated in Fig. 5. The curve of MoS_2_–PDA-48 shows a characteristic band at 3378 cm^−1^, which could be ascribed to the stretch vibrations of –NH_2_, –NH–, and –OH.^49–55^ The characteristic peak at 1607 cm^−1^ could be deduced as a C=C stretching vibration in the benzene ring. At the same time, the weak peaks around 1285 and 876 cm^−1^ could be assigned to C–OH stretching vibration from the catechol groups and C–O stretching, respectively, which indicate that the surface of MoS_2_ was successfully modified by a PDA layer.^56–59^ In addition, the XPS analysis (Fig. 6) demonstrates the composition of MoS_2_ and MoS_2_–PDA-48. Fig. 6a demonstrates the characteristic peaks in the curve of the MoS_2_–PDA-48 composite, such as Mo (3d), O (1s), C (1s), and N (1s). Additionally, the high resolution XPS spectra are shown in Fig. 6b–f. Compared with pure MoS_2_, the relative intensity of C 1s of MoS_2_–PDA-48 decreased and the O 1s peak increased significantly (Fig. 6b and c). In the meantime, it was clear that the relative intensity of the N 1s of MoS_2_–PDA-48 at 400 eV increased obviously in Fig. 6d. In addition, as shown in Fig. 6e and f, the relative intensity of Mo 3d and S 2p in MoS_2_–PDA-48 was significantly reduced compared with the MoS_2_ nanosheets, which also indicates that the PDA layer was successfully coated onto the surface of the MoS_2_ nanosheets. On the other hand, the relative elemental analysis data based on XPS analysis is shown in Table 2. The atom percentages of Mo and S in the table fell from 13.99% and 27.71% to 3.51% and 9.11%, respectively. At the same time, the atomic percentage of N in MoS_2_–PDA-48 accounted for 7.58%, indicating successful PDA modification on the surface of the MoS_2_ nanosheets.

**Table:** Elemental contents (%) of MoS_2_ and MoS_2_–PDA-48 based on XPS analysis

Samples	Mo [at%]	S [at%]	C [at%]	O [at%]	N [at%]
MoS_2_	13.99	27.71	28.94	26.10	3.26
MoS_2_–PDA-48	3.51	9.11	52.56	27.24	7.58

### 3.2 Adsorption performances

**Fig. 7:**
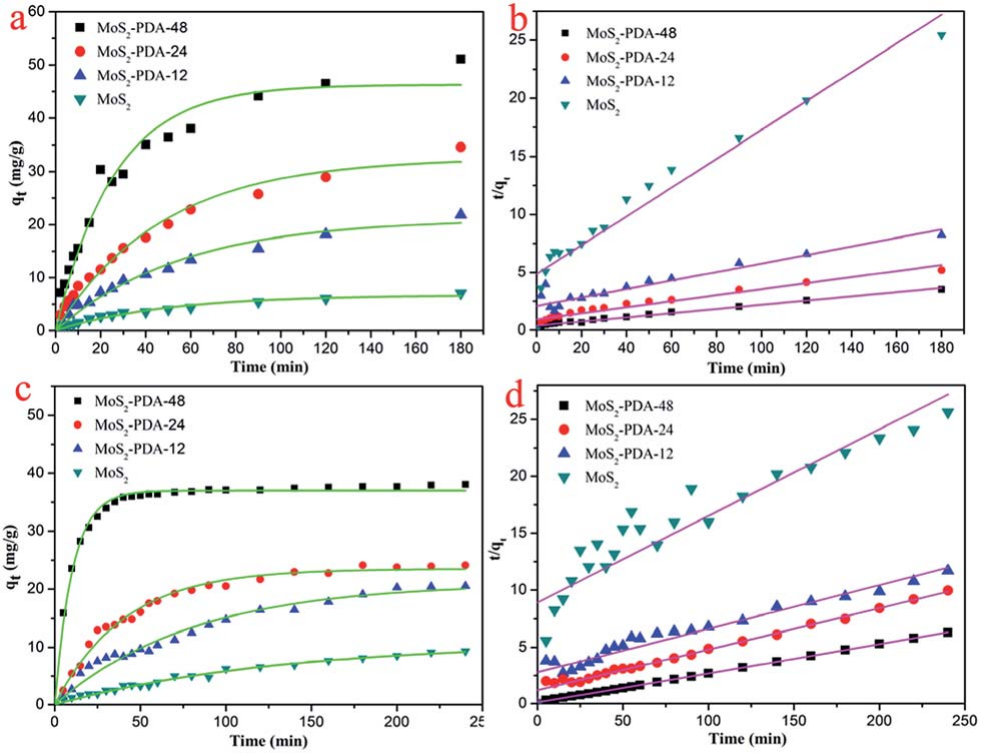
Adsorption kinetics curves of MB (a and b) and ST (c and d) onto MoS_2_ nanosheets and prepared MoS_2_–PDA nanocomposites with different reaction times.

In order to study the adsorption properties of the prepared MoS_2_–PDA nanocomposites, two model dyes (MB and ST) were used in this research work. The concentrations of residual dyes at different reaction time intervals were monitored at different absorbance wavelengths (664 nm for MB; 530 nm for ST). Fig. 7 shows the results of the adsorption kinetics of MoS_2_ nanosheets and MoS_2_–PDA nanocomposites for the removal of MB and ST. It can obviously be observed from Fig. 7a and c that the dye adsorption capacities of the MoS_2_–PDA nanocomposites increased markedly compared to pure MoS_2_ in the equilibrium state, and the adsorption effect of MoS_2_–PDA-48 was the best. The detailed kinetic parameters are shown in Table 3. It should be noted that PDA is a kind of hydrophilic and adhesive material. After PDA anchored onto the surface of the MoS_2_ nanosheets, the core–shell nanostructures formed demonstrated a porous surface with a number of enhanced active sites to improve the adsorption performance of the composites.

Kinetic parameters of the MoS_2_ and MoS_2_–PDA composites for MB and ST adsorption

**Table:** 

MB	Pseudo-first-order model			Pseudo-second-order model		
	*q*_e_ (mg g^−1^)	*R* ^2^	*k*_1_ (min^−1^)	*q*_e_ (mg g^−1^)	*R* ^2^	*k*_2_ (g mg^−1^ min^−1^)
MoS_2_	6.7309	0.9772	0.0208	8.0567	0.9340	0.0032
MoS_2_–PDA-12	21.1223	0.9763	0.0181	27.2183	0.8321	6.46 × 10^−4^
MoS_2_–PDA-24	32.5736	0.9753	0.0212	38.2409	0.9372	7.33 × 10^−4^
MoS_2_–PDA-48	46.2684	0.9665	0.0389	54.9451	0.9801	8.84 × 10^−4^

**Table:** 

Safranine T	Pseudo-first-order model			Pseudo-second-order model		
	*q*_e_ (mg g^−1^)	*R* ^2^	*k*_1_ (min^−1^)	*q*_e_ (mg g^−1^)	*R* ^2^	*k*_2_ (g mg^−1^ min^−1^)
MoS_2_	10.9608	0.9865	0.0077	13.1268	0.8101	6.52 × 10^−4^
MoS_2_–PDA-12	23.4885	0.9878	0.0251	26.2192	0.9231	5.18 × 10^−4^
MoS_2_–PDA-24	21.0367	0.9714	0.0127	27.7239	0.9845	0.0011
MoS_2_–PDA-48	36.9899	0.9929	0.0959	38.610	0.9996	0.0063

In addition, the adsorption kinetic process can be described by classical kinetic models as follows:

The pseudo-first-order model can be represented by eqn (2):2
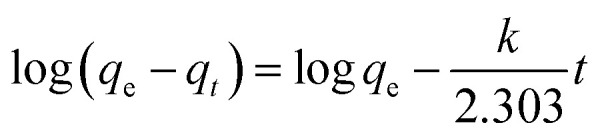


The pseudo-second-order model can be represented by eqn (3):3
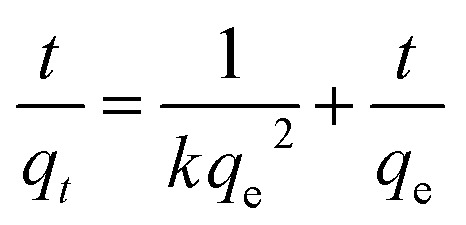
where *q*_e_ is the equilibrium adsorption capacity, mg g^−1^; *q*_*t*_ is the adsorption capacity at time *t*, mg g^−1^; the *k*_1_ and *k*_2_ values are the kinetic rate constants. Kinetic results (Table 3) could be better characterized by a pseudo-first-order model with a correlation coefficient (*R*^2^ > 0.9665) or a pseudo-second-order model with a correlation coefficient (*R*^2^ > 0.8101). Comparing the above adsorption data, it could easily be observed that MoS_2_–PDA-48 showed the best adsorption capacity, which could be related to the rich functional groups on the surface of PDA. And the ability to remove MB seemed higher than that for ST. In addition, the gradual increase in the maximum adsorption indicated that the experimental method used in this study is feasible.

**Fig. 8:**
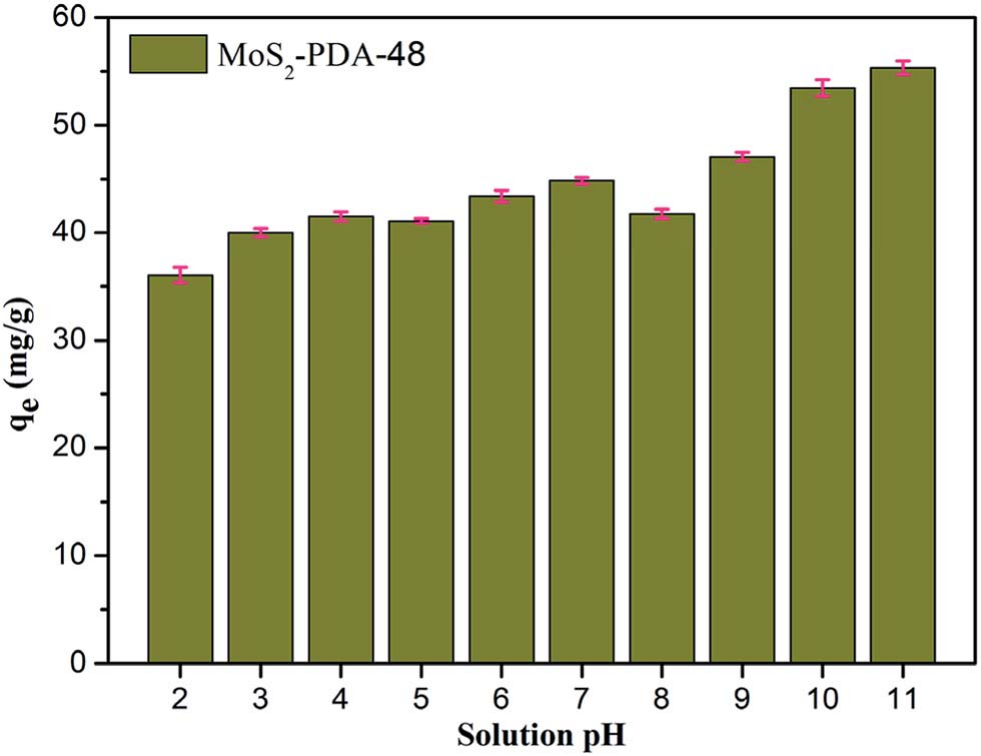
The effect of solution pH on MB adsorption by MoS_2_–PDA-48 nanocomposites.

**Fig. 9:**
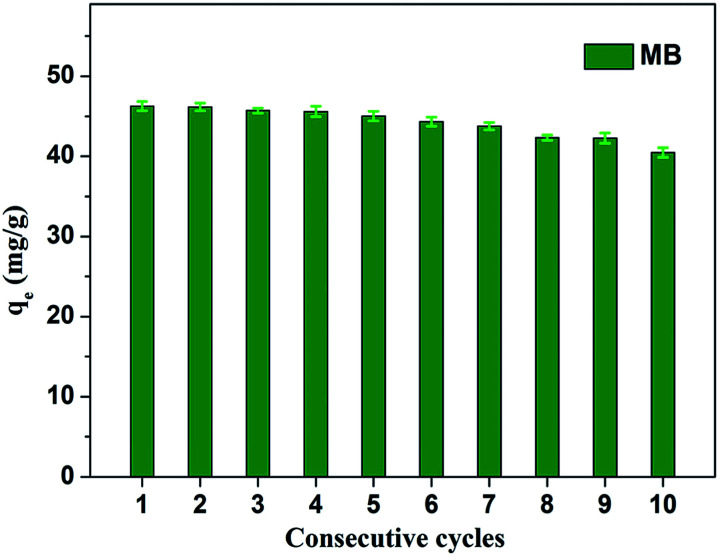
Relative adsorption capacity of MoS_2_–PDA-48 nanocomposite towards MB for different consecutive cycles.

Fig. 8 shows the effect of solution pH on the adsorption of MB to MoS_2_–PDA-48. It can be seen that as the pH of the solution increased from 2 to 11, the adsorption capacity showed an increasing trend from 36.02 mg g^−1^ to 55.32 mg g^−1^. However, this process varied with different pH values. This difference can be presumed to be a result of the protonation of the functional groups on the nanocomposite surface as well as the π–π stacking and electrostatic interactions with the MB molecules.^60^ In addition, in order to demonstrate the reuse of the obtained composites, 10 consecutive cycles were repeated using the same MoS_2_–PDA-48 nanocomposite and fresh MB solution (Fig. 9). This showed that the adsorption capacity of the MoS_2_–PDA-48 nanocomposite still retained a removal rate of 87.4% towards MB and reached 40.45 mg g^−1^. Moreover, Table 4 lists the MB adsorptions of the relevant materials reported in the literature.^61–66^ In contrast, the present prepared MoS_2_–PDA composite materials showed a larger adsorption capacity and eco-friendly preparation process, demonstrating wide application in wastewater treatment and self-assembled core–shell composite materials.

**Table:** Comparison of MB adsorption capacities in the reported literature

Materials	*Q*_max_ (mg g^−1^)	Characteristics	Ref.
Carbon nanotubes	35	Simple process, environmentally friendly	61
GO/Fe_3_O_4_ nanohybrids	32	Poor stability	62
PDA microspheres	90.7	High-efficiency, large adsorption capacity	63
Fe_3_O_4_@MnO_2_ core–shell nanocomposite	9.71	Weak adsorption capacity	64
PVDF/PDA membranes	172.3	Good regeneration ability	65
Fe_3_O_4_@graphene	45.27	Weak adsorption capacity	66
MoS_2_–PDA	46.3	Eco-friendly preparation, good stability	This work

## 4. Conclusions

In summary, we have synthesized core–shell MoS_2_–PDA nanocomposites simply by using mussel-inspired chemistry. A series of characterization techniques demonstrated that the PDA was successfully coated onto the surface of the MoS_2_ nanosheets. The prepared MoS_2_–PDA composites showed effective removal capacities towards two model dyes. The adsorption process was illustrated by pseudo-first-order and pseudo-second-order kinetic models. The present research work is expected to show potential applications in wastewater treatment and self-assembled core–shell composite materials.

## Conflicts of interest

There are no conflicts to declare.

## Supplementary Material
